# Optimization of the Microwave-Assisted Extraction Conditions for Phenolic Compounds from Date Seeds

**DOI:** 10.3390/foods13233771

**Published:** 2024-11-25

**Authors:** Asma Khalfi, María Carmen Garrigós, Marina Ramos, Alfonso Jiménez

**Affiliations:** Department of Analytical Chemistry, Nutrition & Food Sciences, University of Alicante, ES-03690 San Vicente del Raspeig, Alicante, Spain; khalfiasma95@gmail.com (A.K.); mc.garrigos@ua.es (M.C.G.)

**Keywords:** microwave-assisted extraction, polyphenols, date seeds, response surface methodology, antioxidant, chemical composition, functional foods

## Abstract

Date seeds, often discarded during industrial processing, are an underexploited by-product rich in polyphenols with significant antioxidant potential. This study explores the extraction of polyphenols from date seeds using microwave-assisted extraction (MAE) with an organic solvent. The extraction process was optimized using response surface methodology (RSM), focusing on extraction time, ethanol concentration, and temperature. The optimal extraction conditions were 46% (*v*/*v*) of ethanol, at 62 °C and for 27.3 min. Under these optimized conditions, the extraction yield and total phenolic content of the extract are 12.5% and 59 mg gallic acid equivalent g^−1^ of date seed, respectively, as confirmed by the experimental tests. The extract’s antioxidant activity was confirmed through DPPH, ABTS, and FRAP assays. High-performance liquid chromatography with diode–array detection (HPLC–DAD) identified major phenolic compounds, including procyanidin B1, catechin, quercetin-3,5′-di-*O*-glucoside, epicatechin, procyanidin B, and syringic acid, alongside eight other tentatively identified compounds. These findings underscore the potential of MAE as an environmentally friendly technique for producing polyphenol-rich extracts from date seeds, adding value to this by-product and opening avenues for its application in food and nutritional products.

## 1. Introduction

According to the Food and Agriculture Organization of the United Nations (FAO) roughly one-third of food produced for human consumption is lost or wasted globally, which amounts to about 1.3 billion tons per year. In the European Union alone, around 400 million tons of agricultural waste are generated annually. These staggering amounts highlight the scale of this issue and emphasize the urgent need for more efficient waste management and recovery strategies. Such waste represents not only economic losses but also growing disposal challenges and potential environmental pollution. Much of this waste is underutilized despite its significant potential. As a result, there is a strong focus on the recovery, recycling, and upgrading of agri-food wastes. This is especially relevant in the food processing industries, where waste, effluents, residues, and by-products hold considerable recovery potential and can be transformed into higher-value products [1].

Date seeds are a waste product (or a by-product) derived from the technological transformations of date fruits. They represent around 10–15% of date fruit fresh weight and they are used for animal feed or discarded as waste [2,3]. However, this by-product from the date fruit industry has become an environmental problem for the growing and processing areas where it is cultivated. Since the world production of dates reached around 10 million tons in 2022, approximately 1.5 million tons of date seeds are produced every year [4].

Some works have demonstrated the antioxidant and antimicrobial potential of date seeds, which have been linked to treatments against hypertension, coronary heart disease, and high cholesterol levels, while also supporting gut microbiota health [5,6]. These attributes make date seeds valuable ingredients in food applications, where they can act as emulsifiers, stabilizers, and potential substitutes of other antioxidants in different formulations [7]. Date seeds have been reported to contain significant amounts of fiber (75–80%), fats (10–12%), and proteins (5–6%), along with bioactive compounds, such as polyphenols, flavonoids, and tannins. These last compounds are valued not only for their potent antioxidant properties and ability to neutralize free radicals but also for their broad health benefits, which include anti-inflammatory, cardioprotective, and anti-carcinogenic effects [8,9]. In that sense, polyphenols derived from date seeds offer good opportunities to enhance the circular bioeconomy, especially as functional ingredients and natural additives in food, supporting Goal 2 of the United Nations Sustainable Development Goals eliminating hunger by protecting food from oxidation and microorganisms [10].

The valorization of date seeds through polyphenol extraction not only provides a sustainable approach to reduce the environmental impact of agricultural waste, but also adds economic value by creating new potential ingredients for functional foods and nutraceutical products [11,12]. Exploring novel extraction technologies provides efficient, environmentally friendly alternatives to traditional methods, which often require large solvent volumes, risk degrading thermally sensitive compounds, and are time consuming. Concurrently, solid–liquid (SLE), supercritical fluid (SFE), pressurized liquid (PLE), ultrasound-assisted (UAE), and microwave-assisted (MAE) extraction methods are recognized as environmentally friendly methods for bioactive compounds [13].

Among these techniques, MAE is an excellent alternative to extract bioactive compounds from agricultural residues [14,15,16,17]. MAE is based on the generation of electromagnetic waves that selectively heat the sample, inducing plant cell swelling, structural changes, and cell rupture, which significantly enhances the extraction rate [18]. Compared to other extraction methods, MAE is highly efficient for extracting bioactive compounds from plant materials by offering significant advantages, such as reduced solvent consumption, minimized waste production, faster extraction rates, and energy savings [19]. In contrast to PLE and SFE, MAE is less time consuming [20].

Several studies have reported the extraction of phenolic compounds from date seeds using green extraction methods [8,10,11,16,21]. It has also been stated that MAE could be used with different solvents for these extractions, including deep eutectic solvents (DES) [16]. However, unlike previous research, this study focuses on optimizing MAE conditions to maximize the recovery of the dominant group of polyphenols in date seeds: the flavan-3-ols that are present as polymeric proanthocyanidins [6]. These compounds are not only powerful antioxidants but may also provide additional health benefits through their interactions with gut microbiota [6]. Research has shown that polymeric polyphenols, especially those found in the bound fraction, play an important role in supporting gut health by interacting with microorganisms in the colon [22]. A comprehensive study by Sirisena et al. (2017) characterized the free and bound polyphenol fractions in Deglet Nour date seeds, identifying compounds such as protocatechuic acid, catechins, epicatechins, procyanidins B1, B2, and A2, caffeoylshikimic acid, and various hydroxycinnamic acid derivatives. These findings highlight the diversity and potential bioactivity of date seed polyphenols, reinforcing the importance of optimizing their extraction for applications in functional foods and nutraceuticals, depending not only on the target polyphenols but also on the date variety [23]. Moreover, this study is a pioneer to apply MAE in combination with response surface methodology (RSM) to optimize the extraction process for these particular polyphenols. This innovative approach contributes to the field of sustainable extraction techniques, aiming at valorizing food by-products for potential health-related applications.

Considering all these previous reports, this study focused on the development of a green extraction process with MAE to optimize the extraction of polyphenols from date seeds, also studying the antioxidant activity and thermal stability of the obtained extracts. RSM was employed to optimize key MAE parameters, providing a robust statistical approach that streamlines the extraction process and minimizes experimental trials. This study represents an innovative effort in extracting polyphenols from both the free and the bound fractions of date seeds using MAE and RSM. Additionally, it serves as an initial step toward developing a cascade approach to fully valorize this food by-product, enhancing its economic and functional value. The insights gained into date seed polyphenols will contribute to the broader field of food waste valorization and bioactive compound extraction, advancing sustainable methods for recovering valuable compounds from underutilized agricultural by-products.

## 2. Materials and Methods

### 2.1. Materials

Methanol, isopropanol, n-hexane, sodium chloride, and sulphuric acid were purchased from Honeywell SL (Badalona, Spain). Petroleum ether, sodium methoxide 25% wt solution in methanol, isooctane, Folin–Ciocalteu reagent (2N), 2,2-diphenyl-1-picrylhydrazyl (DPPH), 6-hydroxy-2,5,7,8-tetramethylchroman-2-carboxylic acid (Trolox), sodium carbonate, sodium acetate, glacial acetic acid, hydrochloric acid, 2,4,6-tripyridyl-s-triazine (TPTZ), ferric chloride hexahydrate, gallic acid, and a certified reference mixture of 37 fatty acid methyl esters (FAMEs) were acquired from Sigma-Aldrich (Madrid, Spain). Ethanol and Kjeldahl mixed indicator were purchased from Panreac (Barcelona, Spain).

### 2.2. Sample Preparation 

Date seeds were kindly provided by El Monaguillo Alimentación company, situated in El Campello, Alicante, Spain. Firstly, pits were removed from the Deglet Noor date seeds and freeze-dried for 24 h (Telstar LYOQUEST—55 PLUS, Terrassa, Spain). The shells were further milled into particle sizes (<1 mm) using a Vibrating Cup Mill PULVERISETTE 9 (Frisch GmbH, Oberstain, Germany). Since the standards applied by the Technical Association of the Pulp and Paper Industry (TAPPI) recommend to finely adjust the particle size of the raw material to fall within the 0.25–0.40 mm range for optimal determination of its chemical composition, the 1 mm powder was ground by using a Retsch 2000 mill (Haan, Germany) equipped with a mesh with a pore size of 0.4 mm, yielding the appropriate fraction needed for material characterization [24]. The final dried seed powder (DSP) was stored under vacuum at room temperature.

### 2.3. Proximate Composition Analysis 

The proximate composition of the date seeds (moisture, fat, ash, proteins, and carbohydrates) was analyzed in triplicate according to the Association of Official Agricultural Chemists (AOAC) methods. Briefly, moisture content was measured by drying the samples at 105 ± 5 °C until a constant weight was reached. Crude protein content was determined using an automatic macro-Kjeldahl distillation and titration unit (Pro-Nitro-M, JP Selecta, Barcelona) (AOAC 978.04) by calculating the nitrogen content (N × 6.25). Ash content was measured through incineration in a muffle furnace at 600 ± 15 °C (AOAC 923.03), and crude fat content was extracted using the Soxhlet method with petroleum ether for 8 h (AOAC 920.85). The extractives content (TAPPI T204 cm-97), acid-insoluble lignin (TAPPI T222 om-02), as well as the holocellulose and α-cellulose contents, were also analyzed [10]. The hemicellulose content was determined by subtracting the weight of α-cellulose from the total holocellulose content [24,25,26].

Mineral analysis was determined using inductively coupled plasma–mass spectrometry (ICP–MS). Approximately 0.2 g of DSP was acid-digested with 3 mL of HNO_3_ and 1 mL of H_2_O_2_ using an SRC microwave digestion oven (UltraWAVE™, Milestone, Sorisole, Italy). Digestion conditions were as follows: 5 min at 25 °C up to 100 °C, 15 min from 100 °C to 170 °C, 10 min from 170 °C to 240 °C, and 15 min at 240 °C isotherm, finally returning to 30 °C. The digested sample was diluted to a final volume of 13 mL using Milli-Q ultrapure water and filtered through a 0.22 μm nylon membrane. The mineral content of DSP was analyzed in triplicate by the inductively coupled plasma–mass spectrometry (ICP–MS) system (Optima 7300 DV, PerkinElmer, Inc., Shelton, CT, USA) with the following operational settings: 1300 W radiofrequency power, 15 L min^−1^ of plasma flow, 2.0 L min^−1^ of auxiliary gas flow, 0.8 L min^−1^ of nebulizer gas flow, crossflow nebulizer, cyclonic spray chamber, and a sample uptake rate of 1.5 mL min^−1^. Calibration standards were prepared using a multi-element solution at 1 mg L^−1^ (23 elements in diluted nitric acid, Merck, Darmstadt, Germany).

The thermogravimetric analysis (TGA) of DSP was conducted in triplicate using a Mettler Toledo TGA/SDTA 851e instrument (Schwarzenbach, Switzerland). Approximately 5 mg of the sample was placed in an alumina crucible, and it was heated from 25 °C to 700 °C at 10 °C min^−1^ under a nitrogen atmosphere (flow rate of 50 mL min^−1^).

### 2.4. Microwave-Assisted Extraction (MAE)

The extraction of polyphenols from DSP was carried out by using a FLEXIWAVE™ microwave oven (Milestone srl, Bergamo, Italy). A total of 4 g of DSP was carefully weighed into a 100 mL round-bottom flask and filled with 80 mL of the ethanol:water solvent (1:20 sample:solvent ratio). The extraction process was carried out under controlled MAE conditions, which were defined by the Box–Behnken design, as discussed below. Extraction was conducted with varying extraction time, temperature, and ethanol concentration. A power setting of 1200 W was maintained for all experiments, which proved adequate to attain the temperatures required for an efficient extraction. After MAE, samples underwent centrifugation (10,000 rpm), the supernatant was separated, and the resulting liquid extracts were concentrated in a vacuum rotary evaporator (Rotavapor R114, accompanied by a water bath B480, Büchi, Flawil, Switzerland). Water was removed from the extracts using a freeze dryer (Telstar LYOQUEST-85 PLUS). Finally, the resulting samples, named EX_poly_, were stored in a freezer at −20 °C before undergoing further analysis.

### 2.5. Box-Behnken Experimental Design (BBD)

A three-level Box–Behnken design (BBD) was used to explore the impact of extraction parameters, such as temperature (30, 55, and 80 °C), extraction time (5, 22.5, and 40 min), and ethanol concentration (20, 47.5, and 75% *v*/*v*), on two response variables, i.e., extraction yield and total phenolic content (TPC). Five central point replicates were introduced to assess the experimental error. The levels of the experimental design were established based on experimental constraints, the relevant literature, and preliminary tests built upon previous findings from our research group (Table 1). All experimental runs were performed randomly to minimize the effect of unexpected variability in the response variables. The range of the studied independent variables was selected based on preliminary experiments, experimental limitations, constructive characteristics of the used equipment, and the literature. The following second-order polynomial model was applied to the regression analysis of the experimental data (Equation (1)):

Y = β_o_ + ∑β_i_X_i_ + ∑β_ii_X_i_^2^ + ∑β_ij_X_i_X_j_ (1)

where Y denotes the predicted response variable, while X_i_ and X_j_ refer to the independent variables. The constant coefficient is represented by β_0_, and the regression coefficients for the linear, quadratic, and interaction effects are denoted by β_i_, β_ii_, and β_ij_, respectively. The model’s ability to accurately predict experimental data was assessed using the lack-of-fit test and the coefficient of determination (R^2^). The statistical significance of the model parameters was evaluated at a 5% probability level (α = 0.05), and graphical analysis was employed to illustrate the main effects and interactions of the independent variables on the observed responses.

### 2.6. Characterization of Extracts

#### 2.6.1. Extraction Yield

The overall extraction yield (%) was determined using a response variable in the BBD by using Equation (2):

(2) $$Extraction\;yield\left(\%\right)=\frac{W_{ext}}{W_{0}}\times 100$$

where W_ext_ is the weight of the extracted material, and W_0_ is the weight of DSP used for the extraction.

#### 2.6.2. Total Phenolic Content

The Folin–Ciocalteu method was used to quantify the total phenolic content (TPC), and the experimental procedure was carried out according to Coseteng and Lee [27], with some modifications. In brief, 2.5 mL of ×10 diluted Folin–Ciocalteu reagent was added to 0.5 mL of EX_poly_ and vigorously vortexed for 3 min. Subsequently, 2 mL of a 7.5% sodium carbonate solution (*w*/*v*) was added to the mixture, which was then incubated for 30 min in the dark at room temperature. Following incubation, the absorbance was recorded at 756 nm. The total phenolic content (TPC) was determined using gallic acid as the reference standard, and the results were expressed as milligrams of gallic acid equivalent per gram of dry matter (mg GAE g^−1^ DSP).

#### 2.6.3. Antioxidant Activity

The antioxidant activity of EX_poly_ was determined, in triplicate, by using three different spectrophotometric methods: DPPH (2,2-diphenyl-1-picrylhydrazyl), ABTS (2,2′-azinobis-(3-ethylbenzothiazoline-6-sulfonic acid) diammonium salt) assays, and FRAP (ferric-reducing antioxidant power). A short description of each method is given below.

##### DPPH Radical Scavenging Activity

The free radical scavenging activity was determined by the DPPH method using a previously described procedure with some modifications [28]. The absorbance at 517 nm of the fresh DPPH solution was adjusted to 0.900 using a Biomate-3 UV/Vis spectrophotometer (Thermo-spintronic, Mobile, AL, USA). After that, 0.1 mL of EX_poly_ ethanolic solutions at different concentrations was mixed with 2.0 mL of the fresh DPPH solution. The mixture was vortexed and incubated for 30 min in the darkness at room temperature. After that, the absorbance was measured at 517 nm. Trolox was used as standard, with a calibration curve in the 25–250 mg kg^−1^ range (6 points, R^2^ = 0.9991), and results were expressed as mg of Trolox per g of DSP. The DPPH radical scavenging activity (% of inhibition) was calculated by using Equation (3):

(3) $$Inhibition\left(\%\right)=\frac{A_{control}-A_{sample}}{A_{control}}\times 100$$

where A_control_ is the absorbance of the control solution and A_sample_ is the absorbance of each sample.

##### ABTS Assay

The ABTS assay was employed to assess the radical scavenging activity, following the method outlined by Stämpfli et al. with slight modifications [29]. A fresh ABTS^•+^ stock solution was prepared by combining a 7 mM ABTS stock solution with 2.45 mM potassium persulfate. The mixture was allowed to sit in the dark for 16 h to generate the ABTS^•+^ radical solution. Ethanol was then added in the required amount to adjust the absorbance of the ABTS^•+^ solution to 0.700 at 765 nm. In total, 100 µL of the diluted EX_poly_ in ethanol was mixed with 2.9 mL of the diluted ABTS solution. Then, the sample was incubated at room temperature in the dark for 6 min, and the absorbance reduction was measured at 765 nm. The inhibition (%) was calculated analogously to the DPPH method using Equation (3). Trolox was used as the standard, using a calibration curve in the range of 25–250 mg kg^−1^ (6 points, R^2^ = 0.9996), and results were expressed as mg of Trolox per g of DSP.

##### FRAP Method 

The FRAP method was determined following the method proposed by Ebner et al. [30] with some modifications. The FRAP reagent was prepared by combining 0.3 mol L^−1^ of acetate buffer (pH 3.6), 10 mmol L^−1^ of TPTZ dissolved in 40 mmol L^−1^ of HCl, and 20 mmol L^−1^ of aqueous FeCl_3_ in a 10:1:1 (*v*/*v*/*v*) ratio. Then, 0.1 mL of the sample solution in ethanol (35% *v*/*v*) was mixed with 3 mL of the FRAP working solution and incubated at 30 °C for 30 min. The absorbance was measured at 593 nm. Trolox was used as the standard using a calibration curve in the range of 25–250 mg kg^−1^ (6 points, R^2^ = 0.9977), and results were expressed as mg of Trolox per g DSP.

#### 2.6.4. Phenolic Profile 

The phenolic compounds present in EX_poly_ were identified using HPLC–MS. An Agilent 1100 HPLC system, equipped with a quaternary solvent delivery system and coupled to an ion-trap mass spectrometer with an electrospray ionization (ESI) source (Agilent Technologies, Palo Alto, CA, USA) was employed for this analysis. The chromatographic separation was carried out on a HALO C18 column (250 mm × 4.6 mm I.D. × 5 µm) at 30 °C. The mobile phase was consisted of the following: solvent A: MilliQ water + 0.1% formic acid; solvent B: acetonitrile + 0.1% formic acid. The gradient elution program was a linear gradient from 5% to 30% of B for 40 min up to 50% of B in 10 min followed by a linear gradient decreasing to 5% of B in 10 min. The flow rate was 1 mL min^−1^, and the injection volume was 40 µL.

EX_poly_ and standard solutions of phenolic compounds (Procyanidin B1, Syringic acid, Procyanidin B2, epicatechin, and quercetin-3,5′-di-*O*-glucoside) were freshly prepared in ethanol (46% *v*/*v* in water) and filtered through a 0.22 μm nylon membrane before injection.

After identification by HPLC–MS, a quantitative analysis was performed using HPLC–DAD. An Agilent 1260 Infinity Quaternary LC HPLC system (Agilent Technologies, Palo Alto, CA, USA), equipped with a diode array detector (DAD), was employed for this purpose, with simultaneous monitoring at 280, 320, and 360 nm. The experimental conditions were the same as those used previously with HPLC–MS. Quantitative analysis was performed using the external calibration method based on the preparation of calibration curves at seven concentration levels for the aforementioned standard compounds in ethanol (46% *v*/*v* in water), using representative standards of the phenolic compounds, including Procyanidin B1, syringic acid, Procyanidin B2, catechin, epicatechin, and quercetin-3,5′-di-*O*-glucoside. Results for each target phenolic compound present in the EX_poly_ were expressed in equivalents of the respective standard. All analyses were performed in triplicate. To validate the analytical method, the linearity, limit of detection (LOD), limit of quantification (LOQ), and precision (RSD) were assessed.

#### 2.6.5. Thermal Stability 

The thermal stability of EX_poly_ was studied by TGA using a method proposed by Condurache et al. with some modifications [31]. It was based on submitting the sample to repeated thermal cycles at temperatures ranging from 80 °C to 130 °C. The selection of temperature and time parameters was aimed at mimicking conditions relevant to thermal processes used by food and pharmaceutical industries. In brief, 10 mg of sample per 1 mL of water was prepared, adjusting the solution to pH 4. Then, 2 mL of each solution was placed in glass tubes and subjected to a temperature range from 80 °C to 130 °C at 10 °C intervals with 10 min at each temperature, which was controlled using a digital heating block (Techne Dri-Block DB-3D Heating block, Sigma-Aldrich). Once the thermal treatment was completed, the glass tubes were cooled by immersion in iced water to prevent further degradation. Subsequently, the thermally treated extracts were analyzed by the determination of the total phenolic content (TPC) using gallic acid as a reference compound. The results were expressed as mg of gallic acid equivalent per g of extract.

### 2.7. Statistical Analysis

All experiments were conducted in triplicate, with results presented as the mean ± standard deviation (SD). The BBD results were generated and analyzed using Statgraphics Centurion XVI software (Statistical Graphics, Rockville, MD, USA). A graphical analysis of the main effects and interactions between variables was used for results interpretation. The statistical significance of the model parameters was evaluated at a 95% confidence level (α = 0.05). Spearman’s test was used to determine correlations between the obtained data.

## 3. Results and Discussion

### 3.1. Compositional Analysis 

The main results on the compositional study of the DSP are reported in Table 2. Moisture content obtained for DSP was 6.7 ± 0.6 wt%. In a study by Bouaziz et al., the moisture content obtained for date seeds was slightly higher (8.02 ± 0.18 wt%) [32], but these differences may be attributed to the varieties, in particular by the cultivar’s origin and soil. The differences observed for the same cultivar are mainly due to climate particularities, harvesting periods, drying, and storage conditions [33]. The ash content obtained in this study was 1.12 ± 0.12 wt%, which was fully in agreement with results obtained by Demirbas, who found that ash content was 1.1–1.2 wt% [34]. This low ash content is appropriate for chemical reactions involving extraction [35]. Protein and fat contents were 5.5 ± 0.5 wt% and 10.4 ± 1.6 wt%, respectively, while the insoluble lignin content of date seeds was 7.3 ± 0.1 wt%. These results were in general agreement with those reported by several authors [36,37,38]. Results also showed high holocellulose content (50.3 ± 0.2 wt%), which could make DSP a good candidate as a source of cellulose for reinforcement materials and other applications. However, DSP also showed high fat value (10.4 ± 1.6 wt%). This is a relevant result, since fats are one of the potential sources of energy in humans, and consequently, their presence in the diet is essential for health and well-being, as they play vital roles in the body. However, the excessive consumption of fatty foods has significantly contributed to public health issues, such as obesity and cardiovascular diseases, and the environmental impacts of their production deserve significant attention [39]. In this context, valorizing fat-rich waste, such as date seeds, emerges as a fundamental strategy to address sustainable resource management and promote healthier eating habits. The utilization of these waste materials, and the environmental burden associated with their disposal can be alleviated while creating opportunities to integrate nutritious and functional ingredients into the food chain, thereby contributing to developing more sustainable and health-conscious food systems.

On the other hand, Table 3 shows the main results for the mineral content of DSP. Some essential dietary minerals, such as Na, Mg, Ca, Mn, Fe, Cu, and Zn, were identified, all of them developing vital functions necessary for our organism and health [40]. In summary, DSP presents high K content (2022.9 ± 1.2 mg kg^−1^) with 35.95% of the overall mineral content, while P, Cl, and Mg contents were 1449.7 ± 1.2 mg kg^−1^ (25.77%), 629.9 ± 1.3 mg kg^−1^ (11.19%), and 552.1 ± 1.2 mg kg^−1^ (9.81%), respectively. Similar results have been reported by other authors, with the values 26.68% (K), 11.96% (P), 7.25% (Cl), and 1.86% (Mg), respectively [41]. The relative order of concentration in macronutrients was K > P > Cl > Mg, and in micronutrients it was Na > Si> Fe > Zn > Mn.

The analysis carried out with DSP revealed the presence of relevant nutrients and bioactive molecules in its composition. It is a rich source of minerals, proteins, and free sugars, which offer a wide variety of new possibilities to contribute to healthy functional foods.

### 3.2. Thermogravimetric Analysis (TGA) 

**Figure 1:**
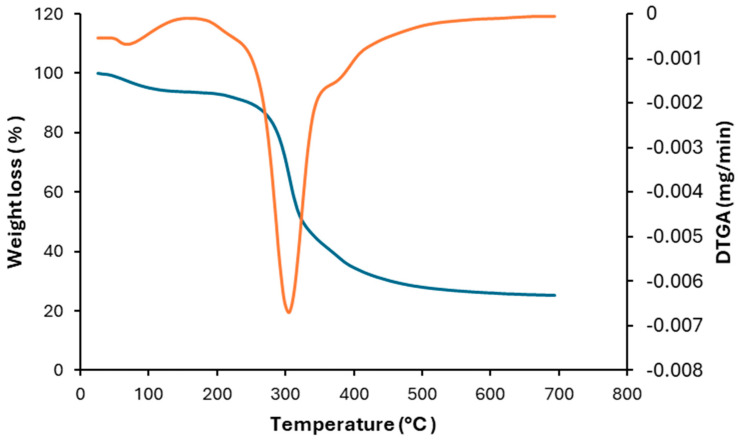
TG (blue) and DTG (orange) curves of DSP.

The thermal stability of DSP was evaluated, and Figure 1 shows the TG and derivative (DTG) curves under an inert nitrogen atmosphere. As usual in TGA studies under continuous non-isothermal heat input, high molecular weight compounds decomposed into lower molecular weight compounds [42].

The thermal degradation of DSP can be considered as a three-step process: dehydration, main thermal degradation, and solid decomposition. The dehydration stage was observed from room temperature to around 100 °C, with a mass loss of around 5 wt% due to residual moisture release and loss of volatile compounds. The second stage corresponds to the main thermal degradation step, occurring from around 250 °C to 500 °C, resulting in a mass loss of around 75 wt%. It should be highlighted that this second step corresponding to the main thermal degradation of DSP can be divided into two parts: The first stage, observable between 200 °C and 350 °C, represents the thermal degradation of the main constituents of DSP (proteins and carbohydrates). In this region, it should also be noted that the decomposition of cellulose, hemicellulose, and a small amount of lignin occurred. The DTG showed that the maximum mass loss occurred at 314 °C, very close to the main peak of the cellulose degradation (around 310 °C) [43]. The second stage in this main degradation peak, between 350 °C and 500 °C, corresponding to the lipids decomposition, showed approximately 10 wt% of mass loss. The last stage observed in this thermogram corresponded to the degradation of carbonaceous structures formed in previous stages [26]. The unburned char (ash) constituted around 21 wt% of the raw DSP. A study proposed that this decline primarily resulted from the breakdown of cellulose and hemicellulose, alongside partial degradation of lignin forming short carbon chains [44].

### 3.3. Microwave-Assisted Extraction Optimization

The MAE conditions were optimized using a BBD design with a total of 17 experimental runs, evaluating the influence of three independent variables: temperature, ethanol concentration, and extraction time. Table 4 presents the results for extraction yield and TPC, which ranged from 9.39% to 12.30% and 33.4 to 58.9 mg GAE g^−1^ for dry powder, respectively. The experiments performed at the central points (runs: 4, 8, 13, 14, and 16 in the experimental design) yielded average values of 11.6 ± 0.4% (RSD = 3%) for extraction yield and 56.2 ± 2.9 mg GAE g^−1^ for DSP (RSD = 5%) for TPC, both of which showed acceptable RSD values and good agreement with the model proposed based on experimental data.

Through multiple regression analysis, quadratic polynomial empirical equations (Equations (4) and (5)) were derived to describe the relationship between each response variable and the independent variables, with A, B, and C representing ethanol concentration, extraction time, and temperature, respectively.

Yield = 9.14168 + 0.124968 A + 0.049249 B − 0.0369971 C − 0.00155333 A^2^ − 0.000538145 AB + 0.000219551 AC + 0.000078903 B^2^ − 0.000270218 BC + 0.00040216 C^2^ (4)

TPC = 4.82623 + 1.19723 A + 0.543519 B + 0.545422 C − 0.0138922 A^2^ + 0.00229228 AB + 0.00341598 AC − 0.0137224 B^2^ − 0.00014885 BC − 0.00680209 C^2^ (5)

An analysis of variance (ANOVA) was performed to assess the reliability of the model and to determine the impact of the studied variables on the selected responses (Table 5). Results demonstrated acceptable coefficients of determination (R^2^) for both yield and TPC, with values of 0.9278 and 0.8736, respectively. The adjusted R^2^ values were closely aligned with the experimental results, confirming the model’s accuracy and the strong correlation between experimental and predicted outcomes. Furthermore, these values validated the precision of the fitting Equations (4) and (5) in correlating with the experimental data.

Additionally, the high *p*-values obtained for lack-of-fit (0.6027 for yield and 0.1485 for TPC) indicated that this parameter was not statistically significant, confirming the models’ adequacy. Finally, the coefficients of variation (CV), which ranged between 0.8730 and 0.9278, reflected the good reproducibility of results, further supporting the model’s reliability.

**Figure 2:**
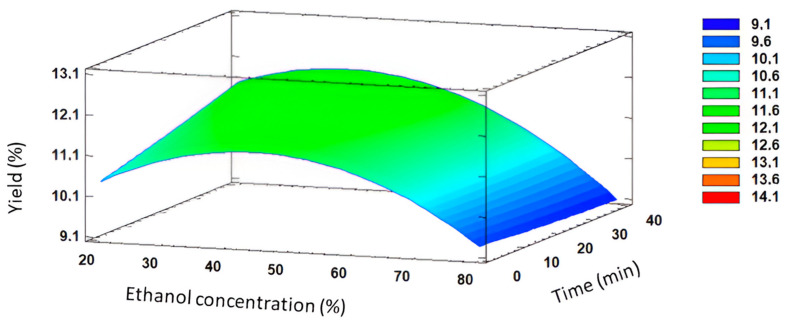
Response surface plot of significant interactions between independent variables on yield (% of ethanol concentration vs. time).

The extraction yield ranged from 9.39 to 12.29 g of EX_poly_ per 100 g of DSP under the 17 experiments (see Table 4). In addition, it was observed that, according to Table 5, ethanol concentration had significant effects (*p* < 0.05) on the overall extraction yield, being the most significant factor for the optimization of MAE for DSP. Moreover, the quadratic effect (AA) was also significant (*p* < 0.05). A three-dimensional surface plot (Figure 2) was achieved through MAE to visualize the independent variable’s influence on yield. Indeed, the influence of ethanol concentration was confirmed in the 3D response surface plot. Results demonstrated the initial increase in yield with rising ethanol concentration levels at a consistent temperature, followed by a maximum value at around 45% (*v*/*v*) and a subsequent decline at higher ethanol concentrations. This observable trend highlighted a pattern wherein an intermediate ethanol concentration level appears optimal for maximizing yield in the DSP extraction.

In general terms, higher yields were obtained at low–medium ethanol concentrations, with a further decrease at higher values. Solvent composition directly relates to the type and quantity of compounds extracted from raw materials. In fact, a low ethanol concentration may encourage the co-extraction of additional substances together with polyphenols and, consequently, enable greater efficiency in the extraction since yield results do not recognize the identity of the extracted compounds. Nevertheless, it is important to acknowledge that extraction yield values do not significantly influence polyphenol extraction performance and the consequent antioxidant activity of the whole extract. This is due to the possibility of the presence of other chemicals, besides polyphenols, with antioxidant capacity in the extracts and polyphenols or other compounds with minimal or no antioxidant activity [45]. According to Chang et al., adding more water to the extraction solvent would increase polarity, which may have contributed to the extraction of polysaccharides and other polar components [46].

On the other hand, TPC obtained after the 17 extractions with different conditions carried out in the BBD varied from 36.0 to 58.9 mg GAE g^−1^ DSP (see Table 4) and reached a maximum at moderate time, temperature, and ethanol concentration. The highest value of TPC with MAE was achieved at 47.5% (*v*/*v*) ethanol concentration, for 22.5 min, and at 55 °C, as determined by the BBD.

**Figure 3:**
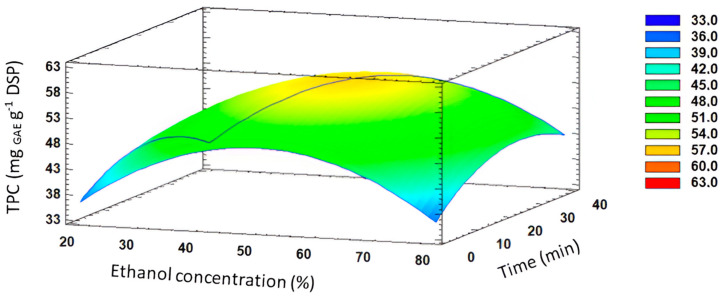
Response surface plot of significant interactions between independent variables on TPC (% of ethanol concentration vs. time).

ANOVA analysis (Table 5) showed that TPC was significantly (*p* < 0.05) influenced by the ethanol concentration, with positive effects by the decrease in solvent polarity. Moreover, three quadratic effects (AA, BB, and CC) also showed significant effects (*p* < 0.05). In this sense, the data in Table 5 illustrate the impact of varying extraction conditions on yield and TPC. Figure 3 illustrates the interaction between ethanol concentration and extraction time, specifically highlighting the combination of 47.5% (*v*/*v*) ethanol and 22.5 min, yielding the maximum TPC value. Regarding the yield of polyphenols, there was a slight enhancement with increasing extraction time from lower to higher levels. However, it decreased after 25 min due to the thermolabile character of polyphenols, resulting in decomposition after long treatments. Based on the observed behavior, it can be concluded that the indirect effect of time and temperature may be considered less significant compared to solvent polarity.

The solubility of phenolic compounds is strongly related to their chemical nature and polarity [47]. Polyphenols are often more soluble in organic solvents than water [48]. Feki et al. reported similar findings, indicating that the ethanol/water ratio exhibited a curvature effect on TPC in Jojoba (*Simmondsia Chinensis*) seed cakes [49]. These authors reported that ethanol-water mixtures facilitated the extraction of a broad spectrum of polyphenols, encompassing not only polar compounds but also weakly polar and apolar compounds. However, at higher ethanol percentages, the solvent may lead to the dehydration of vegetable cells, complicating the diffusion of polyphenols from the plant matrix into the extracting liquid, consequently reducing extraction yields.

### 3.4. Optimal Extraction Conditions

The objective of the previous optimization study was to achieve the highest polyphenol content on EX_poly_ from DSP using MAE while staying within the specified range of extraction parameters. The results from different experimental runs (Table 5) not only support the effectiveness of the optimized conditions but also illustrate the consistency of the data, confirming the robustness of the applied methodology. After analyzing all experimental results and performing statistical analysis, numerical optimization was conducted to determine the optimal level of the three independent variables that would yield the desired response goal, which was determined with a 95% confidence level.

In this study, optimal conditions were obtained for all responses (Table 6): an ethanol concentration of 46% (*v*/*v*), an extraction time of 27 min at 62 °C. Data in Table 6 demonstrated the predicted versus experimental values, underscoring the reliability of the model. The response variables resulting from the multiresponse optimization, specifically extraction yield and TPC, were 11.75% and 55.2 mg GAE g^−1^ of DSP, respectively. The experimental results did not significantly deviate (*p* > 0.05) from the predicted values in all cases. The residual values were analyzed to support the adequacy of the model for extraction (Equation (1)) related to the responses. This analysis revealed a clear connection between the optimized extraction parameters and the resulting yields of phenolic compounds. The interactions between temperature, time, and ethanol concentration play a crucial role in maximizing both yield and TPC, as indicated by the statistical significance of the model parameters.

In conclusion, the predicted values closely aligned with the experimental outcomes obtained under the optimal extraction conditions as confirmed by the response surface methodology (RSM) model, as shown in Table 6.

Subsequent verification experiments were conducted under these optimal conditions in triplicate, yielding experimental results for extraction yield, TPC, DPPH, FRAP, and ABTS as follows: 11.7 ± 0.2%, 49.49 ± 0.11 mg GAE g^−1^ of DSP, 108.6 ± 1.6 mg_trolox_ g^−1^ of DSP, 113 ± 4 mg_r+Trolox_ g^−1^ of DSP, and 74 ± 3 mg_Trolox_ g^−1^ of DSP, respectively. Furthermore, the entire EX_poly_ extraction and characterization process demonstrated high reproducibility, with variation coefficients ranging from 3% to 5% for all the analyzed response variables.

The total phenolic content (TPC) and antioxidant capacity obtained under optimized extraction conditions were either comparable to or higher than those reported in previous studies for date seed extracts. For instance, the TPC value of 49.49 ± 0.11 mg GAE g^−1^ of DSP was higher than those reported in other studies [50,51,52], such as 2983 ± 91 mg GAE 100 g^−1^ obtained for Mistrello et al. [50], and aligning with typical ranges reported for studies using MAE and other advanced extraction techniques. For instance, Li et al. reported a maximum TPC of 997 mg GAE 100 g^−1^ using supercritical water extraction (SCWE) on date residues, which is clearly lower than the values obtained in this study [51]. This comparison supports the efficiency of MAE under optimized conditions to enhance phenolic yield. Further comparisons with other extraction methods emphasize the efficiency of aqueous ethanol in MAE. Pourshoaib et al. demonstrated that aqueous ethanol mixtures produced higher phytochemical yields compared to acetone-based solutions; a finding consistent with the use of aqueous ethanol as a solvent in MAE in this study to maximize phenolic recovery [52].

Al-Farsi and Lee optimized the extraction of phenolic compounds and dietary fibers from date seeds using conventional methods [3]. However, MAE not only matches these yields, but also offers faster extraction times and better preservation of thermally sensitive compounds, illustrating the practical advantages of MAE over traditional methods.

Advanced extraction techniques have also been investigated to optimize the recovery of bioactive compounds from date seeds. Ghafoor et al. showed that supercritical and subcritical CO_2_ extraction was effective to obtain phenolic compounds with notable antioxidant activity [21]; meanwhile, Kehili et al. employed MAE with DES to efficiently extract phenolics from defatted date seeds. The results of this study align with these findings, further confirming MAE as a green and efficient technique to obtain phenolic-rich extracts [16]. However, additional advantages are presented here through the use of response surface methodology (RSM) to optimize extraction conditions, offering greater precision, efficiency, and scalability in phenolic recovery.

In conclusion, the proposed quadratic models proved to be adequate for optimizing the extraction of bioactive compounds from date seeds within the studied experimental range, suggesting a strong correlation between experimental data and the predicted values. These results suggest that the developed models could be effective in predicting the studied response variables in other experimental extraction conditions. Furthermore, microwaves can be considered a sustainable and economically feasible approach that balances efficiency and environmental considerations, resulting in a valuable method for the valorization of bioactive compounds from date seeds.

### 3.5. Thermal Stability

**Figure 4:**
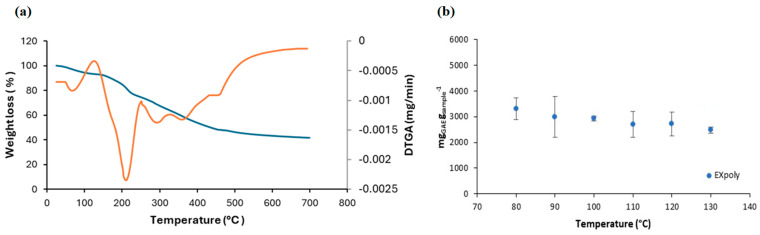
(**a**) TGA (blue) and DTG (orange) curves from EX_poly_ and (**b**) TPC in date seed extracts treated at different temperatures.

Regarding the thermal stability of EX_poly_, the TGA and DTG curves (Figure 4a), obtained under nitrogen atmosphere, revealed three decomposition stages: dehydration (I) (around 80–100 °C), main degradation (II) (around 200 °C), and decomposition of carbonaceous residues (III) (around 300–500 °C). The first zone (I) corresponds to the removal of moisture and the loss of low-molecular-weight volatile compounds. The second degradation zone (II) is associated with the thermal breakdown of organic compounds, primarily due to the degradation of bioactive components within the extract. Finally, the third zone (III) represents the decomposition of carbonaceous solids, indicating the final breakdown of thermally resistant structures within the extract. A similar profile was obtained in pomegranate peel extracts [53] and polyphenol extracts from de-oiled rice bran [54]. Engozogho Anris et al. related the second stage with the degradation of condensed tannins [55] and confirmed the results of this work.

Figure 4b shows the evolution of the total phenolic content (TPC) in EX_poly_ when subjected to heating from 80 °C to 130 °C, as indicated in Section 2.6.5. Results demonstrated that TPC values did not show statistically significant differences (*p* > 0.05) across the studied temperature range.

These experiments were carefully designed to assess the extracts’ thermal stability under conditions simulating common processes in the food industry, such as pasteurization, sterilization, and other high-temperature operations. Results showed that the stability and resilience of phenolic compounds was maintained even at 130 °C for 10 min, suggesting that EX_poly_ retains its antioxidant potential even when exposed to moderately high temperatures, making it suitable for various industrial applications where thermal processing is involved.

### 3.6. Phenolic Profile Using HPLC–MS Analysis

Phenolic compounds represent a class of bioactive compounds commonly found in date seeds, as already demonstrated by the high TPC levels in DSP extracts. These include flavonoids, phenolic acids, and tannins. Previous studies revealed that elevated concentrations of total polyphenols in date seeds render them a notable reservoir of these beneficial bioactive compounds [56,57]. These polyphenols contribute to the antioxidant properties and potential health benefits associated with date seed consumption. In recent years, there has been significant research interest in the phenolic compounds present in dates, driven by their widely recognized health-promoting properties. However, most studies have concentrated on the fruit itself, with limited available information on the phenolic profiles and contents of date seeds.

**Figure 5:**
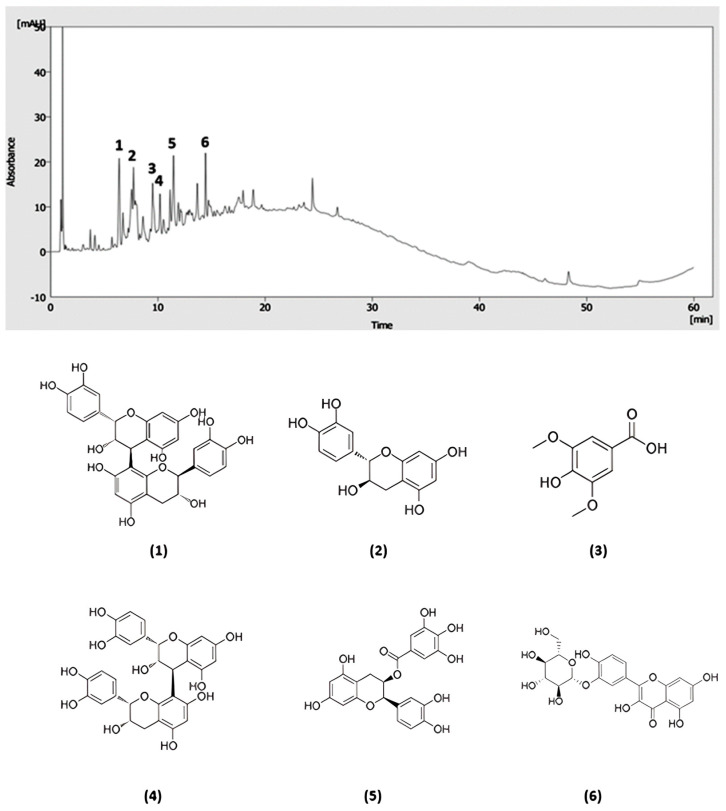
Chromatogram and chemical structures of the six identified compounds by HPLC–MS in EXpoly. (**1**) Procyanidin B1; (**2**) catechin; (**3**) syringic acid; (**4**) procyanidin B2; (**5**) epicatechin; (**6**) quercetin-3,5′-di-*O*-glucoside.

Figure 5 shows the chromatogram and the main compounds identified by HPLC–MS in the EX_poly_ obtained under optimal MAE conditions. Polyphenols from both the free and bound fractions of date seeds were identified, in particular the following: procyanidin B1, syringic acid, procyanidin B2, epicatechin, catechin, and quercetin-3,5′-di-*O*-glucoside. All of them were quantified by HPLC–DAD. The calibration curves of these compounds were obtained using standards at six concentration levels (see Table 7), showing acceptable levels of linearity in their determination coefficients (R^2^), from 0.9904 to 0.9976. The obtained LOD and LOQ values ranged from 4.51 to 9.95 mg kg^−1^ and 13.7 to 30.1 mg kg^−1^, respectively. Finally, the precision in terms of repeatability was evaluated by analyzing standard solutions in triplicate for all concentration levels within the same day. It showed relative standard deviations ranging from 0.11 to 2%, showing good repeatability.

The quantification results showed that procyanidin B1 was the main component in DSP, accounting for 75.8 ± 0.2 mg per 100 g of EX_poly_. According to Hilary et al., procyanidins are part of the dominant group of polyphenols in date seeds. Procyanidins B1 and B2 are flavan-3-ols and belong to the class of condensed tannins, specifically existing as polymeric proanthocyanidins in DSP [6]. Other major components found in DSP were catechin, quercetin-3,5′-di-*O*-glucoside, epicatechin, and procyanidin B2, with a concentration of 43.8 ± 0.4, 42.9 ± 0.1, 37.1 ± 0.2, and 30.0 ± 0.7 mg per 100 g of EX_poly_, respectively. Another compound was syringic acid, with a concentration of 13.8 ± 0.1 mg per 100 g of EX_poly_. 

It is noticeable that all polyphenol concentrations determined in this study were higher than those obtained by Sirisena et al. [23], who used solid phase extraction (SPE) and reported values for polyphenols with at least one order of magnitude lower than those obtained in this study.

It should also be highlighted that date seeds exhibit significantly high levels of total polyphenols compared to other fruits, including grapes, nut seeds, and even date flesh [58,59]. Some reports were published linking the health-beneficial effects of the aforementioned phenolic compounds to anti-inflammatory, antioxidant, anticancer, hypoglycemic, anti-aging, and cholesterolemia activities [60]. Furthermore, previous studies showed that date seeds contain higher levels of tannins compared to the fruit itself [6,60,61]. These tannins, specifically non-hydrolysable condensed tannins, are formed through the condensation of flavan-3-ol monomers, such as catechin and epicatechin, with their concentration varying considerably across different date cultivars. For example, the commercial Deglet Nour cultivar exhibited the highest tannin content (202 ± 10 mg g^−1^ of DSP), while the Hessa variety showed the lowest value (24.2 ± 1.1 mg g^−1^ of SP). In fact, tannins represent the predominant form of phenolic compounds found in date seeds.

The phenolic composition is closely associated with the antioxidant activity observed in the extract, suggesting that these compounds play a crucial role in its functionality. To the best of our knowledge, this is the first study to report the identification and quantification of procyanidin B2 in date seeds, although it has been previously identified and quantified in other plant-based products [62]. Procyanidin B2 is a B-type proanthocyanidin with a structure of (−)-Epicatechin-(4β→8)-(−)-epicatechin [63]. In addition, procyanidins are an important class of bioactive polyphenolic compounds that have gained significant attention for their potential health benefits, based on their antioxidant and anti-inflammatory properties, contributing to the prevention and management of chronic metabolic disorders, such as cancer, diabetes, and cardiovascular diseases [64]. It has been suggested that the health benefits associated with date seeds may be linked to their intricate phytochemical composition, especially the presence of phenolic compounds known for their ability to combat free radicals and oxidative processes [65]. Therefore, the possibilities offered by MAE to maximize the extraction of these compounds from date seeds is a relevant advance to produce functional foods with these important characteristics.

### 3.7. Correlation Analysis

The relationship between total phenolic content (TPC) and antioxidant activities (DPPH, ABTS, and FRAP) has been correlated by using the Spearman analysis. This approach provided valuable insight in relation to the concentrations of the six identified polyphenols in date seed extracts (Section 3.5). These results revealed a strong positive correlation between TPC and the concentration of specific polyphenols, such as procyanidin B1, procyanidin B2, and epicatechin (r = +1.000), suggesting that these compounds are major contributors to the antioxidant performance of the extract. These polyphenols are well-documented for their high radical scavenging abilities and reducing power [64], as well as the antioxidant effectiveness of procyanidins and catechins, especially at high concentrations [66,67]. This relationship supports the effectiveness of the MAE process in getting concentrated fractions of these key antioxidant compounds, thereby maximizing the extract’s antioxidant activity.

Further analysis revealed interesting patterns in these correlations across different assays. For instance, both ABTS and DPPH showed strong positive correlations with TPC. On the other hand, TPC showed a strong negative correlation with syringic acid (r = −1.000), while ABTS and DPPH, which are radical scavenging assays, demonstrated negative correlations with quercetin (r = −1.000). Similarly, the FRAP assay, which measures reducing power, showed a negative correlation with catechin (r = −1.000). These results suggest that while compounds like procyanidins and epicatechin contribute positively to the extract’s antioxidant activity, other phenolics may interact differently within the matrix, possibly due to their structural characteristics or limited concentration in the extract.

It is important to note that in complex matrices, such as date seed extracts, antioxidant capacity often results from synergistic interactions between multiple compounds rather than the sum of the effects of individual polyphenols [68]. Thus, while procyanidins B1, B2, and epicatechin play a predominant role in antioxidant performance, the presence of other polyphenols might influence this activity through unique interactions within the extract. The strong positive correlations observed in this study support the view that targeting specific compounds with MAE can help to maximize the antioxidant potential of date seed extracts, making them promising candidates for functional food applications. All correlations studied here were statistically significant, with *p*-values below 0.05, reinforcing the robustness of these findings. These results fulfill the aim to identify key antioxidant contributors in date seed polyphenols.

## 4. Conclusions

Date seeds, a significant by-product generated in date processing, represent a promising bioresource to obtain valuable compounds and chemicals. This study demonstrates that microwave-assisted extraction (MAE) enhances the extraction efficiency of polyphenols and improves the antioxidant activity of the resulting date seed extracts. MAE conditions were optimized using response surface methodology (RSM), with the ideal conditions being 62 °C, for 27 min and an ethanol concentration of 46% (*v*/*v*) in water. Six polyphenols present in the EX_poly_ extract were identified and quantified: procyanidin B1, catechin, quercetin-3,5′-di-*O*-glucoside, epicatechin, procyanidin B2, and syringic acid. The total phenolic content, the antioxidant properties of the extract, and the identification of the main polyphenols highlight the potential use of DSP as a source of bioactive compounds which could be applied as natural preservatives in food products or the cosmetic industry.

These findings underscore the promising potential for the valorization of this agricultural by-product aligning with the circular economy principles, contributing to the reduction of food waste and the environmental impact generated by agricultural waste. The incorporation of date seed extracts as natural antioxidants or functional additives in food products could significantly enhance shelf life and nutritional value, offering exciting prospects for future research and industrial application.

## Figures and Tables

**Table 1:** Range and levels of independent variables for MAE optimization.

Independent Variables	Code	Coded Levels		
		Lower (−1)	Central (0)	Higher (+1)
Ethanol concentration (%, *v*/*v*)	A	20	47.5	75
Extraction time (min)	B	5	22.5	40
Temperature (°C)	C	30	55	80

**Table 2:** Proximate composition of DSP. Results are expressed as mean ± SD, n = 3.

Components	% Composition DSP
Moisture content	6.7 ± 0.6
Ash	1.1 ± 0.1
Extractives	20.9 ± 0.9
Acid-insoluble lignin	7.3 ± 0.2
Holocellulose	50.3 ± 0.2
Hemicellulose	34.7 ± 0.2
Cellulose	15.6 ± 0.3
Crude proteins	5.5 ± 0.5
Fat content	10.4 ± 1.6

**Table 3:** Mineral contents of DSP. Results are expressed as mean ± SD, n = 3.

Mineral Content	mg kg^−1^ DSP
K	2022.9 ± 1.2
P	1449.7 ± 1.2
Cl	629.9 ± 1.3
Mg	552.1 ± 1.2
Na	502.0 ± 0.9
Si	371.6 ± 1.2
Fe	13.9 ± 1.2
Zn	7.6 ± 0.1
Mn	7.4 ± 0.2

**Table 4:** Box–Behnken experimental design matrix and response values obtained for MAE of date seed powder.

Run	Ethanol Concentration (% *v*/*v*)	Extraction Time (min)	Temperature (°C)	Yield (%)	TPC
	(A)	(B)	(C)		(mg GAE g^−1^ DSP)
1	75	22.5	80	10.47	43.8
2	47.5	40	30	12.05	51.5
3	20	40	55	11.26	33.4
4	47.5	22.5	55	11.25	52.1
5	47.5	5	80	11.87	44.2
6	47.5	5	30	11.15	45.3
7	75	22.5	30	9.39	42.3
8	47.5	22.5	55	11.22	58.0
9	20	5	55	10.54	39.9
10	75	40	55	9.78	45.4
11	75	5	55	10.09	47.4
12	20	22.5	30	11.22	43.9
13	47.5	22.5	55	12.11	54.3
14	47.5	22.5	55	11.71	57.8
15	20	22.5	80	11.59	36.0
16	47.5	22.5	55	11.54	58.9
17	47.5	40	80	12.30	50.2

**Table 5:** ANOVA results for yield and TPC of DSP extraction. (A: ethanol concentration; B: extraction time; C: temperature. * Significant, *p* < 0.05).

YIELD					
Source	Sum of Squares	Df	Mean Square	F-Value	*p*-Value
A	3.10	1	3.10	22.99	* 0.0087
B	0.38	1	0.38	2.78	0.1705
C	0.67	1	0.67	4.98	0.0894
AA	5.81	1	5.81	43.11	* 0.0028
AB	0.27	1	0.27	1.99	0.2311
AC	0.09	1	0.09	0.68	0.4571
BB	0.01	1	0.01	0.02	0.8991
BC	0.06	1	0.06	0.41	0.5546
CC	0.27	1	0.27	1.97	0.2328
Lack-of-fit	0.28	3	0.09	0.69	0.6027
Pure error	0.54	4	0.13		
Cor. Total	11.35	16			
R^2^	0.9278				
Adj. R^2^	0.8350				
TPC					
A	82.72	1	82.72	9.98	* 0.0342
B	1.75	1	1.75	0.21	0.67
C	9.63	1	9.63	1.16	0.3417
AA	464.74	1	464.74	56.07	* 0.0017
AB	4.87	1	4.87	0.59	0.4862
AC	22.06	1	22.06	2.66	0.1781
BB	74.36	1	74.36	8.97	* 0.0401
BC	0.02	1	0.02	0.00	0.9661
CC	76.10	1	76.10	9.18	* 0.0388
Lack-of-fit	78.29	3	26.10	3.15	0.1485
Pure error	33.16	4	8.29		
Cor. Total	901.51	16			
R^2^	0.8733				
Adj. R^2^	0.7104				

**Table 6:** Optimal MAE conditions for DSP with predicted and validated values. Results are expressed as mean ± SD, n = 3.

	Ethanol Concentration (%)	Temperature (°C)		Time (min)	
	46	62		27	
Response	Yield	TPC	DPPH	FRAP	ABTS
Experimental results	11.7 ± 0.2	49.49 ± 0.11	108.6 ± 1.6	113 ± 5	74 ± 3
Predicted results	11.7	55.19	-	-	-

Yield is expressed in %, TPC is expressed in mg GAE g^−1^ of DSP, and DPPH, FRAP, and ABTS are expressed in mg_trolox_ g^−1^ of DSP.

**Table 7:** Quantification of phenolic compounds in EXpoly by HPLC–DAD. Results are expressed as mean ± SD, n = 3.

Peak	Compound	Rt *	Calibration Range *	Linearity *	LOD *	LOQ *	RSD *	EX_poly_ *
1	Procyanidin B1	6.40	5.46–106.9	0.9904	8.04	24.4	0.2	75.8 ± 0.2
2	Catechin	7.71	5.39–106.9	0.9946	7.73	23.4	1.1	43.8 ± 0.4
3	Syringic acid	9.45	5.75–109.1	0.9937	9.95	30.1	0.8	13.8 ± 0.1
4	Procyanidin B2	10.23	6.37–105.9	0.9976	5.67	17.1	2	30.2 ± 0.7
5	Epicatechin	11.50	6.47–109.1	0.9976	4.51	13.7	0.11	37.1 ± 0.2
6	Quercetin-3,5′-di-*O*-glucoside	14.45	5.46–106.9	0.9985	4.99	15.1	0.3	42.9 ± 0.2

* Rt: (min); calibration range: (mg kg^−1^); linearity (R^2^); LOD: (mg kg^−1^); LOQ: (mg kg^−1^); RSD: (%); EX_poly:_ (mg 100 g^−1^ DSP).

## Data Availability

The original contributions presented in the study are included in the article, further inquiries can be directed to the corresponding authors.
